# Application of DNA aptamers to block enterotoxigenic *Escherichia coli* toxicity in a *Galleria mellonella* larval model

**DOI:** 10.3389/fchem.2024.1425903

**Published:** 2024-08-29

**Authors:** Maria Margarida Barros, Joana Castro, Daniela Araújo, Ricardo Oliveira, Ana Maria Campos, Sónia Silva, Divanildo Outor-Monteiro, Carina Almeida

**Affiliations:** ^1^ National Institute for Agrarian and Veterinariay Research (INIAV), Vairão, Portugal; ^2^ Veterinary and Animal Research Centre (CECAV), University of Trás-os-Montes and Alto Douro, Vila Real, Portugal; ^3^ Centre of Biological Engineering, University of Minho, Braga, Portugal; ^4^ LABBELS–Associate Laboratory, Braga, Portugal; ^5^ LEPABE—Laboratory for Process Engineering, Environment, Biotechnology and Energy, Faculty of Engineering, University of Porto, Porto, Portugal; ^6^ ALiCE—Associate Laboratory in Chemical Engineering, Faculty of Engineering, University of Porto, Porto, Portugal

**Keywords:** ETEC, F4 fimbriae, aptamers, virulence, *in vivo* blocking, *Galleria mellonella*

## Abstract

Enterotoxigenic *Escherichia coli* (ETEC) is the major bacterial cause of diarrheal diseases in pigs, particularly at young ages, resulting in significant costs to swine farming. The pathogenicity of ETEC is largely dependent on the presence of fimbriae and the ability to produce toxins. Fimbriae are responsible for their initial adhesion to the intestinal epithelial cells, leading to the onset of infection. In particular, the F4 type (K88) fimbriae are often attributed to neonatal infections and have also been associated with post-weaning diarrheal infections. This disease is traditionally prevented or treated with antibiotics, but their use is being severely restricted due to the emergence of resistant bacteria and their impact on human health. Emerging approaches such as aptamers that target the F4-type fimbriae and block the initial ETEC adhesion are a promising alternative. The aim of this study is to assess the effectiveness of two aptamers, Apt31 and Apt37, in controlling ETEC infection in the *G. mellonella in vivo* model. Initially, the dissociation constant (K_D_) of each aptamer against ETEC was established using real-time quantitative PCR methodology. Subsequently, different concentrations of the aptamers were injected into *Galleria mellonella* to study their toxicity. Afterwards, the anti-ETEC potential of Apt31 and Apt37 was assessed in the larvae model. The determined K_D_ was 81.79 nM (95% CI: 31.21–199.4 nM) and 50.71 nM (95% CI: 26.52–96.15 nM) for the Apt31 and Apt37, respectively, showing no statistical difference. No toxicity was observed in *G. mellonella* following injection with both aptamers at any concentration. However, the administration of Apt31 together with ETEC-F4+ in *G. mellonella* resulted in a significant improvement of approximately 30% in both larvae survival and health index compared to ETEC-F4+ alone. These findings suggest that aptamers have promising inhibitory effect against ETEC infections and pave the way for additional *in vivo* studies.

## 1 Introduction

Swine enteric colibacillosis is a disease characterized by an intestinal infection caused by the colonization of enterotoxigenic *Escherichia coli* (ETEC). This infection mostly causes illness or death in neonatal and weaned pigs, making it responsible for significant economic losses worldwide (Castro et al., 2022). Porcine ETEC pathotypes are characterized by the expression of specific fimbriae adhesins that mediate bacterial colonization of the intestinal mucosal surface. When both the immunological systems and the gut microbiota are poorly developed, ETEC colonizes and produce heat-labile (LT) and/or heat-stable (ST, including STa/STb subtypes) enterotoxins that can have local and systemic effects (Barros et al., 2023; Luppi, 2017). Some pathotypes of Shiga toxin (Stx or VT), namely, type 2e (Stx2e), are also frequently found in ETEC strains (Luppi et al., 2016). The most commonly detected fimbriae in ETEC are F4 (previously known as K88) and F18 (García et al., 2020; Luise et al., 2019). Intestinal colonization and disease caused by ETEC depend on F4 or F18-specific receptors. ETEC-F4 is usually associated with post-weaning diarrhea (PWD) in recently weaned piglets occurring 2–3 days after weaning, whereas F18 is commonly found in association with diarrhea 2–6 weeks after weaning (Luise et al., 2019). The age-dependent expression of F4 and F18 receptors in the small intestine may explain why ETEC-F4+ infection mainly occurs immediately after weaning and during the neonatal period, whereas ETEC-F18+ infection mainly occurs later in the post-weaning period (Luppi, 2017; Luppi et al., 2016).

PWD is usually prevented or treated with antibiotics, but, in recent years, their use has been highly restricted due to the growing phenomenon of antimicrobial resistance (Barros et al., 2023). It is therefore important to begin the transition to other, more sustainable, approaches. In this regard, novel strategies such as aptamers, which are small single-stranded oligonucleotides capable of binding to target molecules, seem to be a promising alternative to block the initial adhesion mediated by fimbriae in ETEC. Li and colleagues reported two aptamers, Apt31 and Apt37, for ETEC with F4-type fimbriae capable of specifically detecting these bacteria (Li et al., 2011). The SELEX methodology followed directed the selection towards the fimbriae protein and demonstrated the applicability of the selected aptamers as diagnostic recognition molecules (Li et al., 2011). Although the binding model between the aptamers and the F4 fimbriae has not been studied, the observed interaction means that the aptamers, after binding, have a spatial significance in the potential additional binding of the ETECs. Of note that it has been described that the interaction of aptamers with their targets results mainly from the compatibility of physical structures and/or the stacking of chemical groups stabilized by hydrogen bonds, electrostatic interactions, hydrophobic effects, π-π stacking, van der Waals forces, or combinations of these different forces (Oliveira et al., 2022). Therefore, the physicochemical interaction can block receptor-binding domains of the fimbriae from interacting with receptors and, thus, neutralize their role in the initial adhesion of ETEC strains and consequently the development of infection (Keefe et al., 2010; Zhou and Rossi, 2017).

However, until now the potential of aptamers to block ETEC-mediated infections in animal models has not been evaluated. As a proof of concept, the present study focuses on evaluating the anti-ETEC potential of two aptamers previously described in the literature, Apt31 and Apt37 (Li et al., 2011), using an *in vivo G. mellonella* larval model (Ferreira et al., 2023). It is important to highlight that *Galleria mellonella* larvae have gained attention as an alternative model to study bacterial infections due to their genetic similarity to mammals and their convenience and cost-effectiveness compared to traditional mammalian models (Ménard et al., 2021). Additionally, the immune response of *G. mellonella* shares similarities with mammalian innate immunity, making it a suitable model for evaluating potential therapeutic interventions (Tsai et al., 2016).

## 2 Materials and methods

### 2.1 Strains and culture maintenance

Two ETEC strains, SP11 and SP31, which encompass the most predominant fimbriae linked to PWD, namely, F4 and F18, were included in this study. Strain SP11 encodes F4, along with the enterotoxins STb and LT, while strain SP31 encodes F18 and the enterotoxin Stx2e. Other additional bacterial strains were included herein as controls: *E. coli* strain K12 (wild-type); *Klebsiella pneumoniae* strain ATCC 43816 and *Staphylococcus aureus* strain ATCC 25923. All bacterial strains were grown on Tryptic Soy Agar (TSA) (Liofilchem, Roseto degli Abruzzi, Italy) for 18–24 h at 37°C and cryopreserved at −80°C in cryovials composed of Tryptic Soy Broth (TSB) (Merck, Darmstadt, Germany) and 20% (v/v) of Glycerol (Biochem Chemopharma, Cosne-Cours-sur-Loire, France).

### 2.2 Determination of K_D_ of Apt31 and Apt37 against ETEC-F4+

The dissociation constant (K_D_) was determined for the two selected DNA aptamers (Apt31, Apt37) with proven affinity for the F4-fimbriae type, considering the selection conditions reported by the authors (Li et al., 2011). Their sequence (depicted in Table 1) is composed of two primer sites on both sides with 18 nucleotides and a central portion with 60 nucleotides that distinguish both aptamers. The main objective of this step was to establish a comparison between the reported K_D_ and the one obtained using the qPCR methodology used by our group. Briefly, it was used a binding buffer (BB) containing 138 mM NaCl (Biochem Chemopharma), 20 mM Tris-HCL pH 7.4 (NZYtech, Lisbon, Portugal), 2.7 mM KCl (Merck), 1 mM CaCl_2_ (Scharlau, Barcelona, Spain), 0.5 mM MgCl_2_ (Merck), and 0.1 mg mL^-1^ of DNA salmon sperm (Invitrogen, Waltham, United States), and a washing buffer (WS) composed of BB+ 0.05% (v/v) Tween 20 (VWR, Portland, United States) to guarantee the reported physicochemical conditions to aptamers functionality. At first, 10 nM concentrations (400, 300, 200, 150, 100, 50, 10, 5, 1, 0) of both aptamers (Eurogentec, Seraing, Belgium) were prepared with BB in 1.5 mL low binding microcentrifuge tubes, then heated at 95°C for 5 min, cooled in ice for 10 min, and lastly maintained at room temperature for 15 min. After, the mentioned concentrations of aptamers (100 μL ) were incubated with 700 μL of ETEC strain SP11 bacterial suspension at 10^8^ CFU mL^-1^ in 1.5 mL low binding microcentrifuge tubes for 30 min, at 37°C with a mixing of 500 rpm. Post-incubation, each sample was washed 3 times with WB followed by centrifugation for 5 min at 6,000 rpm to discard the nonbounded ssDNA aptamers. The recovered pellet was resuspended in 100 μ L of ultra-pure water and finally, aptamers were eluted by heating at 95°C for 10 min and recovered in the supernatant by centrifugation for 5 min at 6,000 rpm. Two independent assays were carried out.

**TABLE 1:** Single-stranded DNA aptamers sequence (5′- 3′) of Apt31 and Apt37 according to Li et al., 2011.

ssDNA aptamers	Primer forward region (18 nt)	60-Base central region	Primer reverse region (18 nt)
Apt31	CGTACGGTCGACGCTAGC	ACA​CTC​TTT​TGC​TCG​TGT​TTT​TGC​CTG​TTA​CAT​AAA​ATG​AAT​CAG​TGG​ATG​TTT​CCT​TCT	CACGTGGAGCTCGGATCC
Apt37		GGA​GAC​CGT​ACC​ATC​TGT​TCG​TGG​AAG​CGC​TTT​GCT​CGT​CCA​TTA​GCC​TTG​TGC​TCG​TGC	

To quantify the bound aptamer in the eluted samples, a real-time quantitative PCR (qPCR) methodology using SYBR green was conducted with the following conditions: initial polymerase activation/denaturation (95.0°C, 2 min); 25 amplification cycles (denaturation at 95°C, 5 s; annealing at 60°C, 15 s). At the end of each cycle, a SYBR green read was performed. For this protocol, 20 μL reactions containing 1× NZYSupreme qPCR Green Master (NZYTech), 0.4 μ M primers (forward and reverse as mentioned in Table 1), and 2 μL of DNA template were prepared. All amplification reactions were performed in duplicates, including a no-template control (NTC) in each run to check for contamination. For each qPCR assay, standard concentrations of the aptamers under study were also prepared and quantified using the same PCR conditions. A standard curve was built using quantification cycle (Cq) values vs log(standard concentration) to determine the equation of the line that fits the distribution obtained. At the end, the equation of the linear regression was used to determine the concentration of eluted aptamer and estimate the binding affinity for each aptamer.

The K_D_ was determined by a non-linear regression analysis $(\left|Aptamer\;eluted\right|=\frac{B_{\max\;}\left|Inital\;aptamer\right|}{K_{D}+\left|Initial\;aptamer\right|})$ using GraphPad Prism version 8, GraphPad Software, Boston, Massachusetts United States, www.graphpad.com.

### 2.3 Evaluation of aptamers toxicity in *Galleria mellonella* model

Larval survival experiments were adapted with minor modifications from previous studies (Araújo et al., 2022; Ferreira et al., 2023). The larvae were maintained on a diet of pollen grains and bees wax at 25°C in the darkness. Worms of the last instar, weighing approximately 250 mg, were selected for the following experiments. The toxicity of Apt31 and Apt37 was evaluated by injecting a range of concentrations (1µM, 10 μM, 20 µM). A control of PBS 1× was also included herein. For each condition tested, each larva (n = 10 × 4 groups) was injected with 5 µL of suspension into the haemolymph via the hindmost left proleg, previously sanitized with 70% (v/v) of ethanol, using a micro syringe. After the injections, the larvae were placed in Petri dishes and stored in the dark at 37°C. Survival was monitored for 4 days (each 24 h) and if the larvae did not move in response to touch, they were considered dead. The *G. mellonella* health index, which assesses four main parameters: larval activity, cocoon formation, melanization, and survival, classified with a score determined in a previous study (Loh et al., 2013). First, larval survival is analysed, if the larva is alive it is scored as 2, if it is dead it is scored as 0. In terms of larval activity, larvae are scored between 0 and 3, where three means that larvae show movement without stimulation and 0 means that larvae do not show any movement. Cocoon formation is scored between 0 and 1, where 1 is a full cocoon and 0 is no cocoon. Melanisation is scored between 1 and 4, where 4 is given if the larva retains its original creamy colour and 1 if the larva is completely dark. All experiments were performed in triplicate and at least three independent assays were performed. Ethical approval is not required for the study of this animal model.

### 2.4 Evaluation of the anti-ETEC potential of aptamers in infected *Galleria mellonella*

The effectiveness of each aptamer, Apt31 and Apt37, in controlling ETEC infection was evaluated using also the *G. mellonella* model. For that, ETEC strains, SP11 and SP31, were grown in TSB at 37°C and 120 rpm, and in the exponential phase, the cells were centrifuged, washed, and suspended in PBS (pH 7), and the cell density was adjusted to the final concentration of 10^8^ CFU mL^-1^ in PBS. The cell suspensions were normalized using OD_600nm_ and the CFU. mL^-1^. Other bacterial strains (*E. coli* strain K12; *K. pneumoniae* and *S. aureus*) were also included in the assay as controls, using the same concentration of 10^8^ CFU mL^-1^ in PBS. Per bacterial strain, each larva (n = 10 × 4 groups) was injected with 5 µL of suspension into the haemolymph via the hindmost as described in section 2.3. Of note that each set of larvae was injected with the Apt31 or Apt37 together with each bacterial strain individually (final concentration 500 nM) (Soundy and Day, 2020). As a control negative, one group of larvae was injected with the same volume of PBS only, while another group with bacteria only (positive control). Survival over time and *G. mellonella* health index were evaluated as described in the previous section. To investigate the potential of Apt31 and Apt37 to control the progression of bacterial infection, some larvae infected with the SP11 strain and treated with both aptamers were sacrificed at 48 and 72 h for histological processing. The fat body of each larva was removed by a ventral midline incision with a scalpel blade. The fat bodies were placed in 10% (v/v) neutral buffered formalin (PanRaec AppliChem, Barcelona, Spain) and stored at 4°C for 24 h to fix the structures. Paraffin blocks were sectioned at 4–5 *µ*m using a microtome (Accu-Cut^®^ SRM™, Labometer, Lisbon, Portugal) and the sections were stained with the periodic acid Schiff (PAS) (Bio-Optica Milano spa, Milan, Italy). Tissue sections were observed and photographed using a Leica DM1000 LED microscope coupled to a digital camera (Leica, Microsystems, Wetzlar, Germany).

### 2.5 Statistical analysis

The K_D_ experiments were performed in duplicate with two independent assays, using the following equation, $Y=B_{\max}X/\left(K_{d}+X\right)$ , according to the saturation curve. $B_{\max}$ is the maximum specific binding, $K_{d}$ is the equilibrium dissociation constant, Y is the concentration of bounded eluted aptamer and X is the initial concentration of aptamer incubated with the bacterial cells. These data are expressed as the mean of the four measurements of two independent assays together with 95% confidence interval, assuming a Gaussian distribution. The K_D_ of both Apt31/Apt37 were compared using independent *t*-Test analysis with a confidence level of 95%. The *in vivo* trials using *G. mellonella* model were performed in triplicate and in at least three independent assays. Data are expressed as the mean ± standard deviation (SD) of at least three independent experiments. For the *G. mellonella* model, Kaplan-Meier survival curves were plotted and differences in survival were calculated by using the log-rank Mantel-Cox statistical test. The health index results were compared using ANOVA analysis with Holm-Sidak’s multiple comparisons tests and a confidence level of 95%. All tests were performed using GraphPad Prism 8^®^ (GraphPad Software, CA, United States).

## 3 Results

### 3.1 K_D_ values for Apt31 and Apt37 against ETEC-F4+

**FIGURE 1:**
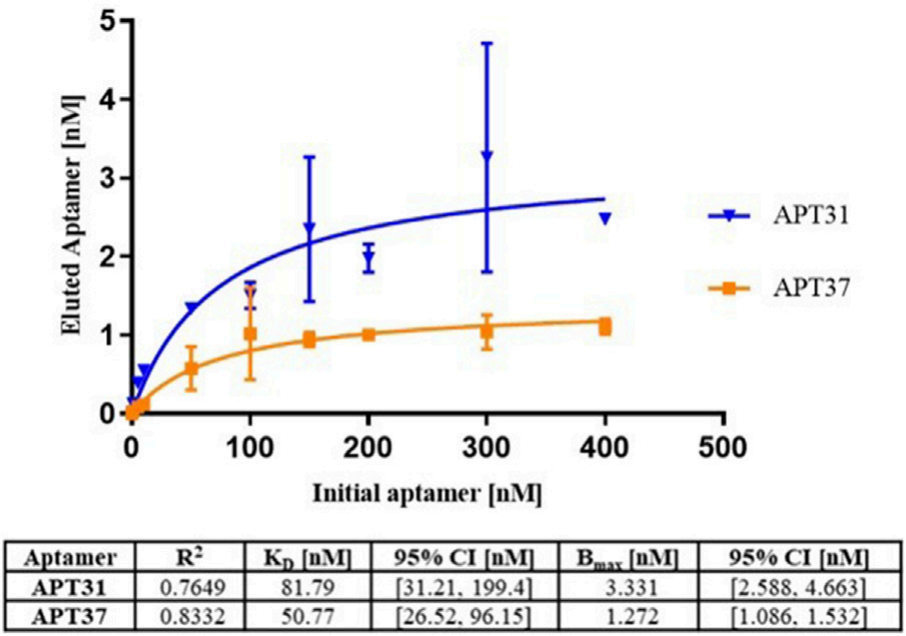
Values of the dissociation constant (K_D_) for Apt31 (represented in blue) and for Apt37 (represented in orange). The information in the table below reports the R-squared (*R* ^2^), dissociation constant (K_D_) and maximum specific binding (B_max_) with the corresponding 95% confidence interval.

The kinetic parameters of the two aptamers, Apt31 and Apt37, with a reported affinity for F4-fimbriae were determined by the qPCR methodology (Li et al., 2011). The binding data for the aptamer concentrations tested (0–400 nM) against a fixed concentration of ETEC strain SP11 suspensions ( ≈ 10^8^ CFU mL^-1^) was fitted to a non-linear regression according to the formula mentioned above (*R* ^2^ = 0.7649 for Apt31 and *R* ^2^ = 0.8332 for Apt37). The determined K_D_ was 81.79 nM (95% CI: 31.21–199.4 nM) and 50.77 nM (95% CI: 26.52–96.15 nM), while B_max_ was 3.331 nM (95% CI: 2.588–4.663 nM) and 1.272 nM (95% CI: 1.086–1.532 nM) for the Apt31 and Apt37, respectively (Figure 1). Since affinity is inversely proportional to the K_D_, the Apt37 seems to demonstrate a higher affinity in relation to Apt31. However, a statistical comparison of the parameters of Apt31 and Apt37 revealed that the K_D_ is not significantly different (*p* > 0.05). Both aptamers were therefore submitted for testing in the *G. mellonella* model.

### 3.2 Assessment of anti-ETEC potential of Apt31 and Apt37 using the *Galleria mellonella* larval *in vivo* model

**FIGURE 2:**
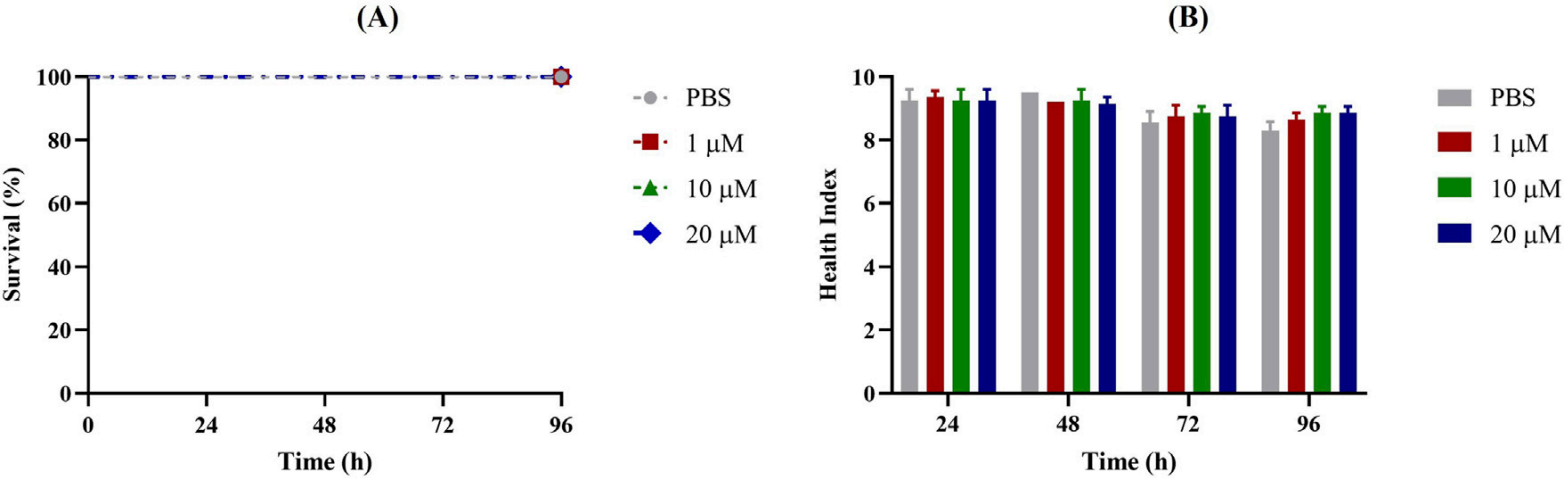
Toxicity of Apt31 of non-infected *Galleria mellonella*. Survival curves **(A)** and health index score **(B)** of *G. mellonella* administrated with 1, 10 and 20 μM of aptamer concentrations. As a negative control, larvae were injected only with PBS.

**FIGURE 3:**
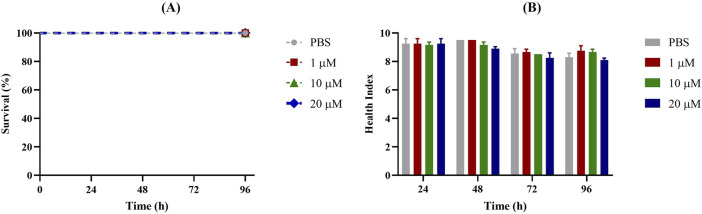
Toxicity of Apt37 of non-infected *Galleria mellonella*. Survival curves **(A)** and health index score **(B)** of *G. mellonella* administrated with 1, 10 and 20 μM of aptamer concentrations. As a negative control, larvae were injected only with PBS.

First, *G. mellonella* larvae were injected with different concentrations of Apt31 and Apt37 to assess their respective levels of toxicity. Based on data shown in Figures 2, 3, no toxicity in the *G. mellonella* model was evidenced for both aptamers evaluated (Apt31, Apt37). In fact, no effect was observed on the larval health index and larval survival up to 96 h post-injection for any of the concentrations of each aptamer tested.

**FIGURE 4:**
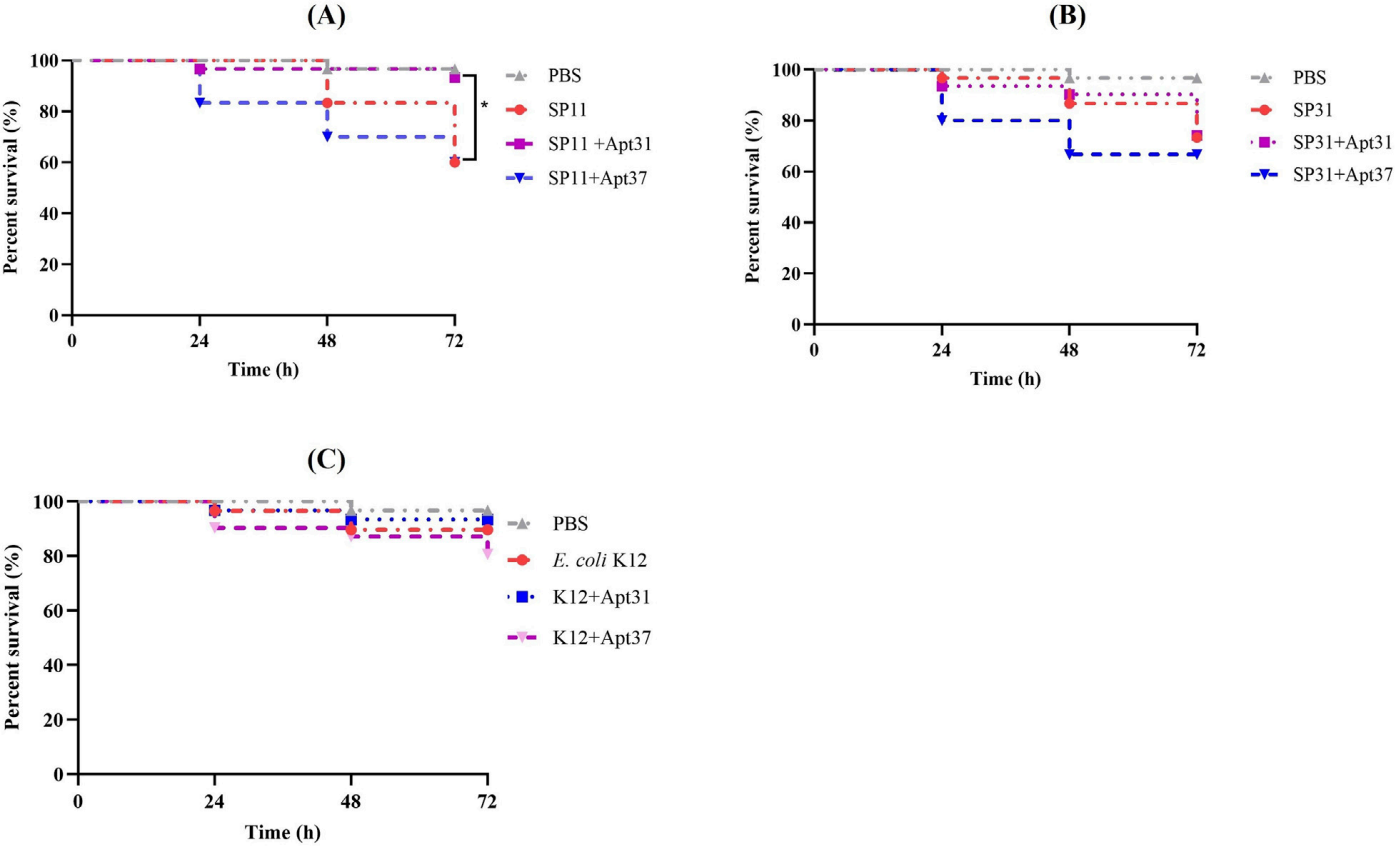
Effect of the DNA aptamers on the survival of infected *Galleria mellonella*. Survival curves of *G. mellonella* treated with 500 nM of Apt31 and Apt37. Larvae were infected with ETEC strain SP11 **(A)**, ETEC strain SP31 **(B)**, *Escherichia coli* strain K12 **(C)**. As a negative control, larvae were injected only with PBS. *Significant difference between positive control (larvae infected only with bacteria) and treated with aptamers (*p* < 0.05).

Afterwards, the *G. mellonella* larvae model was used to study *in vivo* the effects of Apt31 and Apt37 against ETEC-F4+ infection. For that, *G. mellonella* infected with ETEC strains (SP11; SP31) and other bacterial species (*E. coli* K12; *S. aureus*; *K. pneumoniae*) were treated with both aptamers at a concentration of 500 nM. It is important to remark that each set of larvae was injected with the Apt31 or Apt37 together with each bacterial strain individually. Figure 4 shows the results obtained after 72 h of post-infection for 3 *E. coli* strains, two ETECs (Figures 4A, B) and one no-pathogenic strain (Figure 4C). In *G. mellonella* infected with *E. coli* strain SP11 (F4-fimbriae type), it is noteworthy that a single dose of Apt31 enhances larval survival by 33% over 72 h (*p* < 0.05) (Figure 4A). In contrast, when the larvae were treated with Apt37, a slight decrease in larvae survival was observed, but this difference is not statistically different from *G. mellonella* infected with SP11 alone or SP31 alone (Figures 4A, B, respectively). However, such an effect was not due to the toxicity of the aptamer, as it alone does not show any toxicity up to 20 μ M of the concentration, as shown in Figure 3. Furthermore, it is also important to highlight that no significant effect of Apt31 was observed against the other ETEC strain analyzed (SP31) (Figure 4B). In addition, no effect was observed for either aptamer against other *E. coli* (non-ETEC) (Figure 4C), *S. aureus* (Supplementary Figure S1A), and *K. pneumoniae* (Supplementary Figure S1B).

**FIGURE 5:**
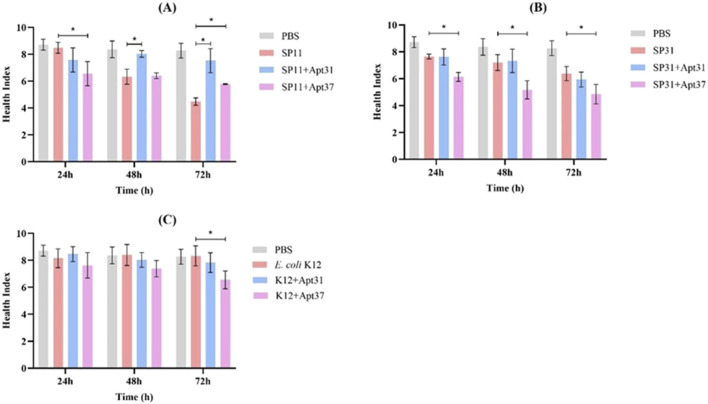
Effect of the DNA aptamers on the health index scores of infected *Galleria mellonella*. Larvae were infected with ETEC strain SP11 **(A)**, ETEC strain SP31 **(B)**, *Escherichia coli* strain K12 **(C)**, and treated with 500 nM of Apt31 and Apt37. As a negative control, larvae were injected only with PBS. *Significant difference among positive control (larvae infected only with bacteria) and treated with aptamers (*p* < 0.05).

The *G. mellonella* health index was also determined, through the evaluation of larval activity, cocoon formation, melanization, and survival. The results are presented in Figure 5. It should be noted that when *G. mellonella* was infected with SP11 and treated with Apt31, the health index increased after 48 h (by around 17%) and 72 h (by around 30%) (*p* < 0.05), which corroborates the observed survival rates (Figure 4). In the case of SP31, when *G. mellonella* was treated with Apt31, no differences were observed compared to the control (Figure 5B). In contrast, with Apt37, a decrease is observed in the larvae health index for all times tested (*p* < 0.05) (Figure 5B). For the wild-type strain, *E. coli* K12, the health index in the larvae injected only with bacteria and with aptamers was similar to the PBS, emphasizing the lower virulence related to this strain (Figure 5C). However, it can be seen that in the case of Apt37, the health index was significantly lower compared to the positive control (*p* < 0.05), revealing that Apt37 does not have a positive effect against *E. coli*. For the other species tested, *S. aureus* and *K. pneumoniae*, the health index in the bacteria control was extremely low. For *S. aureus*, no differences were observed between the aptamers and bacteria control over 72 h of infection (Supplementary Figure S2A). For *G. mellonella* infection with *K. pneumoniae*, no differences were observed between control and aptamer treatment at any time point tested, except at 24 h when Apt37 was used to treat *G. mellonella* infection (*p* < 0.05) (Supplementary Figure S2B).

**FIGURE 6:**
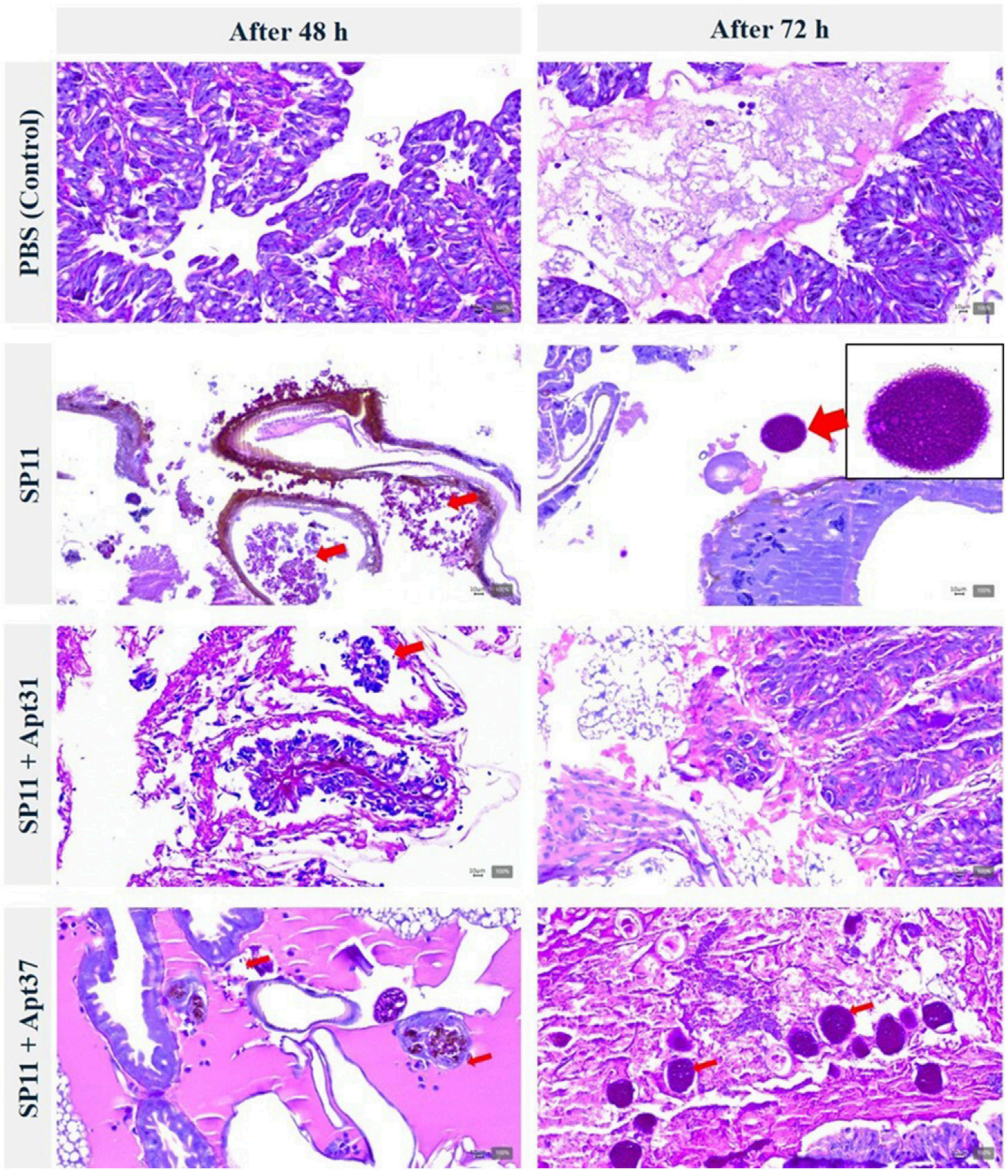
Effect of DNA aptamers on the progression of ETEC SP11 infection in *Galleria mellonella* fat body. Histological images of larvae infected with SP11 (at 48 and 72 h) and treated with Apt31 and Apt37. The larvae sections were labeled with periodic acid Schiff coloration. Red arrows are an example of ETEC colonization. The magnification images were at ×200.

The progression of the effect of the aptamers on SP11 infection was also evaluated by observation of the fat body of *G. mellonella* after histological procedures, as shown in Figure 6. The presence of a bacterial agglomerated system can be observed in the fatty body of SP11-infected larvae after 72 h without aptamer treatment. Additionally, the histological images showed a significant reduction in both the quantity and size of SP11 agglomerates following 48 and 72 h of Apt31 treatment. In the case of Apt37, a huge quantity of SP11 was observed in the fat body after 72 h, which corroborates the survival rates (Figure 4) and health index (Figure 5) results obtained, revealing the lack of efficacy of this aptamer to control the SP11 infection.

## 4 Discussion

The rise of antibiotic resistance among bacterial pathogens, including ETEC, poses a serious threat to public health. Traditional antibiotic treatments for ETEC infections are becoming less effective due to the emergence of resistant strains (Christaki et al., 2020). Therefore, alternative approaches to combat bacterial-associated infections are urgently needed to tackle ETEC-associated morbidity and mortality without contributing to the spread of antibiotic resistance.

Over the last decade, aptamers emerged as a promising tool for diagnostic or even therapeutic purposes (Afrasiabi et al., 2020; Araújo et al., 2024). Aptamers therapeutic effect has been demonstrated for other bacterial pathogens, with promising results that could eventually give rise to new drugs in the future. For instance, aptamers for *S. aureus* (Ommen et al., 2022), *Pseudomonas aeruginosa* (Wang et al., 2018) and *Streptococcus* spp. (Hosseini et al., 2022) have proven that their direct administration and/or aptamer-targeted drug delivery is able to inhibit the formation of biofilms and the development of infections (Araújo et al., 2024). In addition, several aptamers evolved for diagnostic purposes, that target relevant virulence features of bacterial pathogens, might also present potential as therapeutic molecules (Nimjee et al., 2017). This work took advantage of this and selected aptamers previously applied in the detection of F4-fimbriae protein that could also be applied as a biomolecular solution against disease caused by ETEC-F4+ bacteria. Since F4-fimbriae are involved in the initial attachment to the epithelial cell wall of the small intestine, assisting the bacteria in anchoring and proliferating, the aptamers can block the progression of ETEC-F4+ infection by inhibiting the anchoring process (Li et al., 2011).

In this study, we propose to investigate the efficacy of DNA aptamers in blocking ETEC toxicity using the *G. mellonella* larval model *in vivo*. By exploiting the unique properties of aptamers, such as their high binding affinity and specificity, we aimed to selectively interfere with critical virulence factors produced by ETEC, thereby attenuating its pathogenic effects. It is important to highlight that the aptamers used herein, Apt31 and Apt37 were obtained from a SELEX (Systematic Evolution of Ligands by Exponential Enrichment) procedure carried out by Li and colleagues (Li et al., 2011). These aptamers were selected directly for the pure F4-fimbrial protein, and the K_D_ values and specificity were previously reported.

The re-evaluation of the K_D_ values of the aptamers with our methodology against the whole bacterial cell (Figure 1) showed that both aptamers, Apt31 and Apt37, have a high binding affinity with values of 81.79 nM (95% CI: 31.21–199.4 nM) and 50.71 nM (95% CI: 26.52–96.15 nM), respectively. K_D_ of both aptamers (Apt31, Apt37) to these bacterial cells was not statistically different (*p* > 0.05) and did not differ statistically from the reported K_D_ values determined using pure F4 fimbriae, in which Apt31 has K_D_ of 36 ± 8 nM whereas Apt37 has 25 ± 4 nM (Li et al., 2011). Even so, the variance in the K_D_ values between our method and those in the original work may be attributed to the differences in the target employed. The original work used fimbrial protein F4 isolate, whereas we used the whole cell of ETEC-F4 type. Additionally, discrepancies may arise from slight variations in methodology, as well as in biological and physicochemical conditions. It is also important to note that the K_D_ determination methodology used in this study uses whole bacteria as a target, so there is a possibility that part of the aptamer quantified, after elution, was result of internalization and not just binding to the F4-fimbrial protein present on the membrane. Even so, this portion of the quantified aptamer should be reduced, since the direct internalization of nucleic acids in prokaryotic cells, especially in Gram-negative bacteria and unlike eukaryotic cells, is very limited, due to the complexity of the membrane and the lack of internalization mechanisms (Benizri et al., 2019; Kauss et al., 2020; Readman et al., 2017).

The research findings revealed that the Apt31 was the sole candidate to demonstrate a positive impact *in vivo* on the *G. mellonella* model. It increased the survival rate (Figure 4) and improved the health index of larvae (Figure 5) by around 30%, while also reducing the density of cells present through the fat body (Figure 6). These results suggest a potential higher level of pharmacokinetic activity (Nimjee et al., 2017). Regarding Apt37, the results were unexpected, as Apt37 had a more detrimental effect on *G. mellonella* compared to SP11 (Figure 4A) or SP31 alone (Figure 4B). Further research is crucial to uncover the mechanism behind the aptamer-bacterial cell interaction.

As a correlation between Apt31 treatment and improved survival of infected larvae was observed, this study suggests that aptamers may be a promising treatment option for ETEC infections in mammals. Even so, being a DNA aptamer, its stability (half-life) *in vivo* will be reduced (perhaps to minutes), since susceptibility to enzymatic degradation by nucleases present in practically all biological environments is one of the main limitations of the application of aptamers in therapeutic solutions. The future development of an anti-ETEC solution with these aptamers will require post-SELEX improvement with nucleic acid mimics (NAMs) to increase their chemical and biological stability and guarantee their functionality *in vivo*. Even so, this study shows that by targeting key virulence factors, aptamers can effectively neutralize bacterial pathogenicity offering a promising approach for the treatment of ETEC-related diarrheal illness (Ferreira et al., 2023). These findings highlight the potential of aptamers as emerging biotechnological approaches for combating bacterial infections.

## 5 Conclusion

In conclusion, this study highlights the promise of aptamers as a novel inhibitory strategy to control ETEC infections and underscores the significance of using alternative model systems like *G. mellonella* larvae for preclinical evaluation of bacterial agents. Apt31 established a proof-of-concept of the efficiency of aptamers to fight against ETEC in our tested conditions. Nevertheless, further research is necessary for making aptamer endure the gastric passage, being delivered in the intestinal lumen of the piglets. Additionally, investigating the pharmacokinetics and immunogenicity of aptamers in mammalian models will be crucial for evaluating their translational potential as therapeutic agents.

## Data Availability

The original contributions presented in the study are included in the article/Supplementary Material, further inquiries can be directed to the corresponding author.
