# Supplementary material for: Application of DNA aptamers to block enterotoxigenic Escherichia coli toxicity in a Galleria mellonella larval model
Source: Front Chem. 2024 Aug 29;12:1425903. doi: 10.3389/fchem.2024.1425903 (PMC11390681; doi:10.3389/fchem.2024.1425903)
Supplement: Supplementary file 1 [file DataSheet1.docx]

Supplementary Material

**Application of DNA aptamers to block enterotoxigenic *Escherichia coli* toxicity in a *Galleria mellonella* larval model**

## Supplementary Figures


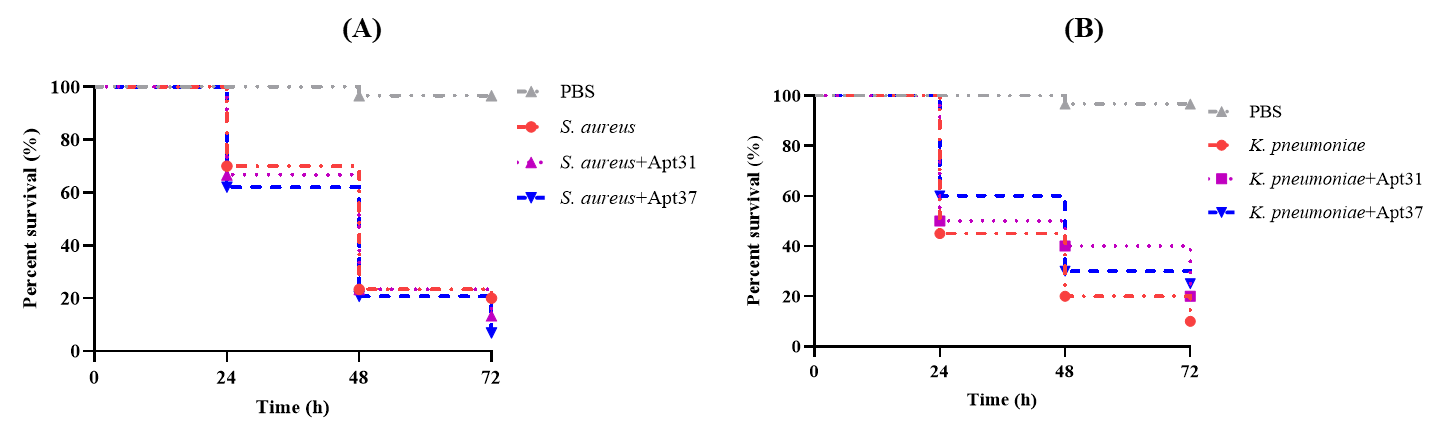


**Figure S1. Effect of the DNA aptamers on the survival of infected *Galleria mellonella*.**  Survival curves of *G. mellonella* treated with 500 nM of Apt31 and Apt37. Larvae were infected with ETEC strain *S. aureus* (A), *K. pneumoniae* (B). As a negative control, larvae were injected only with PBS.


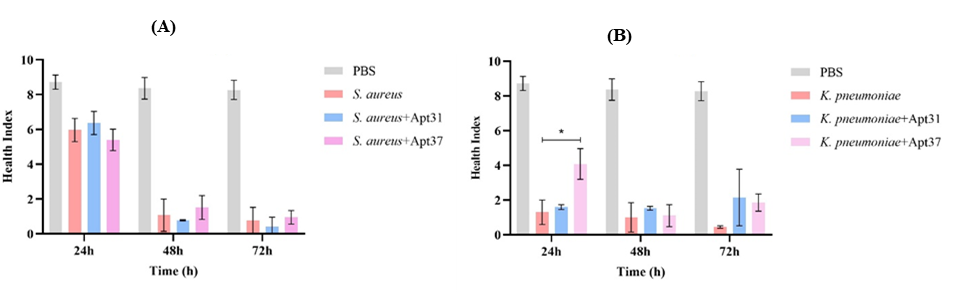


**Figure S2. Effect of the DNA aptamers on the health index scores of infected *Galleria mellonella*.** Larvae were infected with *S. aureus* (A) and *K. pneumoniae* (B) and treated with 500 nM of Apt31 and Apt37. As a negative control, larvae were injected only with PBS. *Significant difference among positive control (larvae infected only with bacteria) and treated with aptamers (P < 0.05).
